# Supplementary figures and images for: Temporal and spatial dynamics in soil acoustics and their relation to soil animal diversity
Source: PLoS One. 2022 Mar 8;17(3):e0263618. doi: 10.1371/journal.pone.0263618 (PMC8903300; doi:10.1371/journal.pone.0263618)

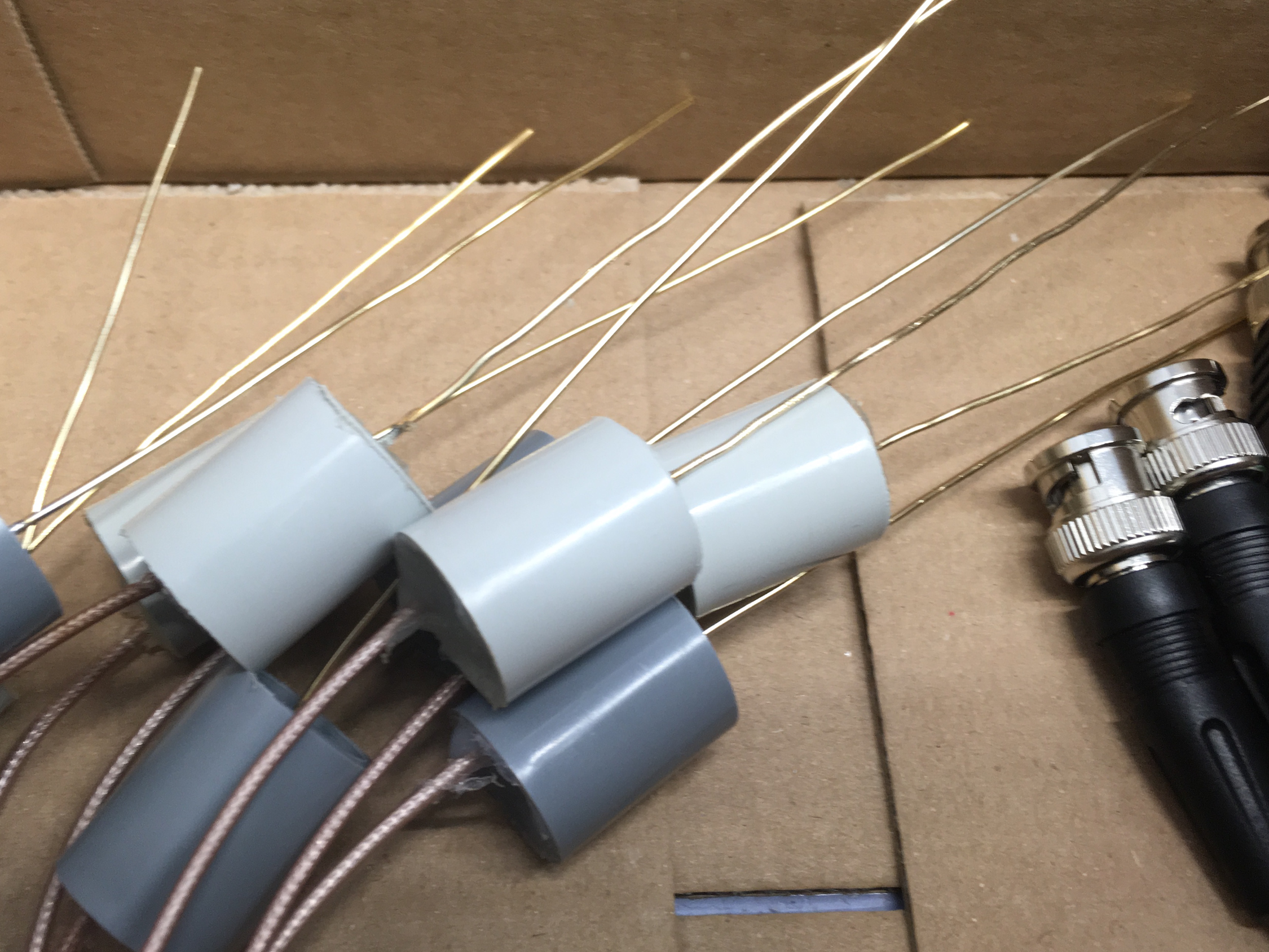

Supplement: S1 Fig — They consist of a piezo diaphragm from Murata that is 15 mm in diameter and 0.2 mm in thickness. A 10 cm long and 1 mm thick gold-plated copper wire needle was soldered to the back of the brass plate of the diaphragm. The needle functions as a waveguide; it catches the acoustic waves in the ground and passes them onto the piezo element. The diaphragm resonates with the captured sound waves and generates electrical voltage in the electrode on the back side, which is amplified and recorded. The contact microphone is surrounded by a protective plastic housing and is insulated against moisture and short circuits with silicone and an epoxy layer directly on the electrode. A 30 cm coaxial cable leads from the sensor to the preamplifier. To record sounds in the soil, the needle is inserted 10 cm deep into the ground. This allows for the detection of sounds within a radius of approximately 30–100 cm. (TIF) [file pone.0263618.s001.tif]

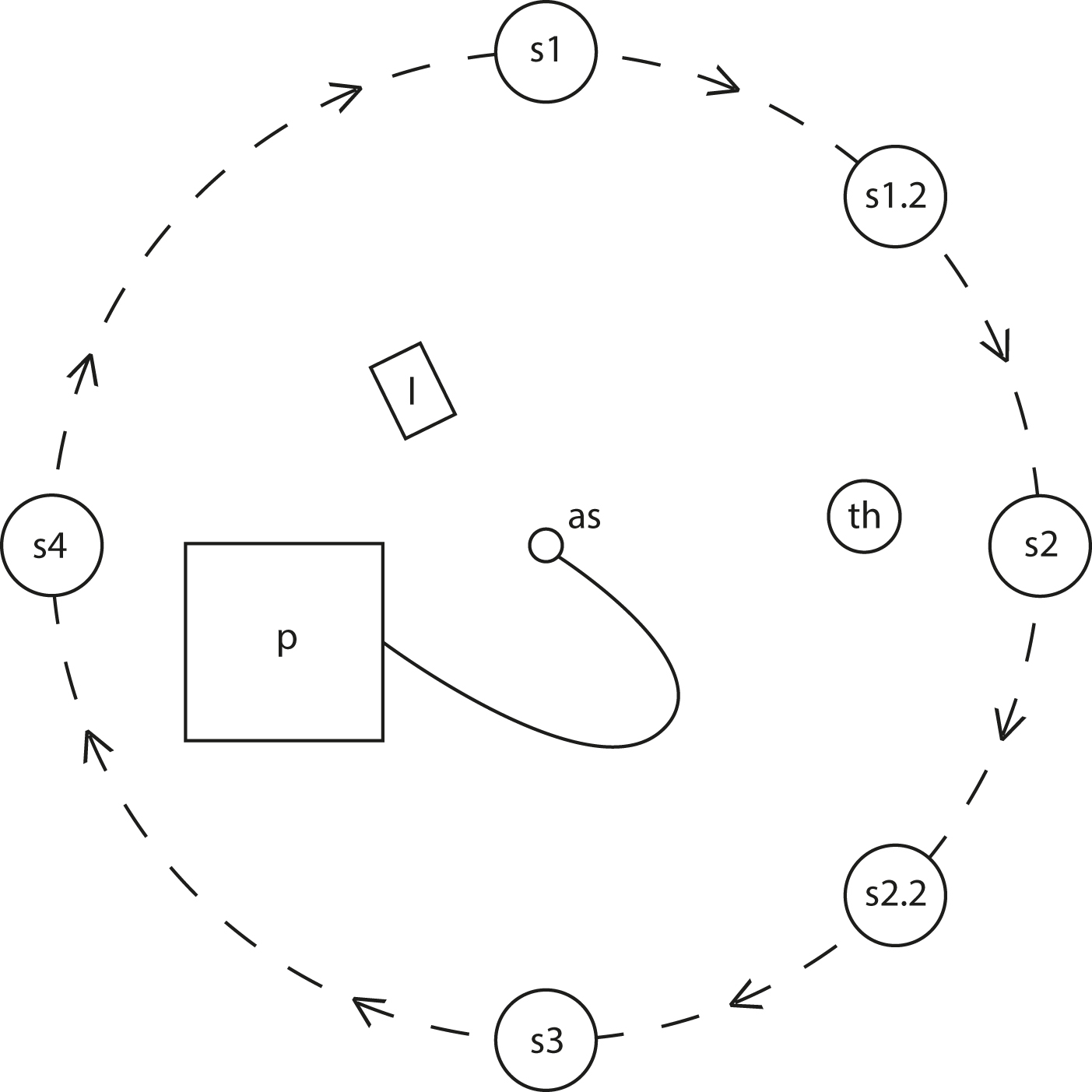

Supplement: S2 Fig — The sampling points were circularly distributed around the acoustic sensor in the middle. Radio of the circle; 50 cm. p = preamplifier, l = light sensor, th = soil temperature and humidity sensor, as = acoustic sensor. Soil samples: s1 = spring 2018, s1.2 = spring 2019, s2 = summer 2018, s2.2 = summer 2019, s3 = fall 2018, s4 = winter 2018. (TIF) [file pone.0263618.s002.tif]

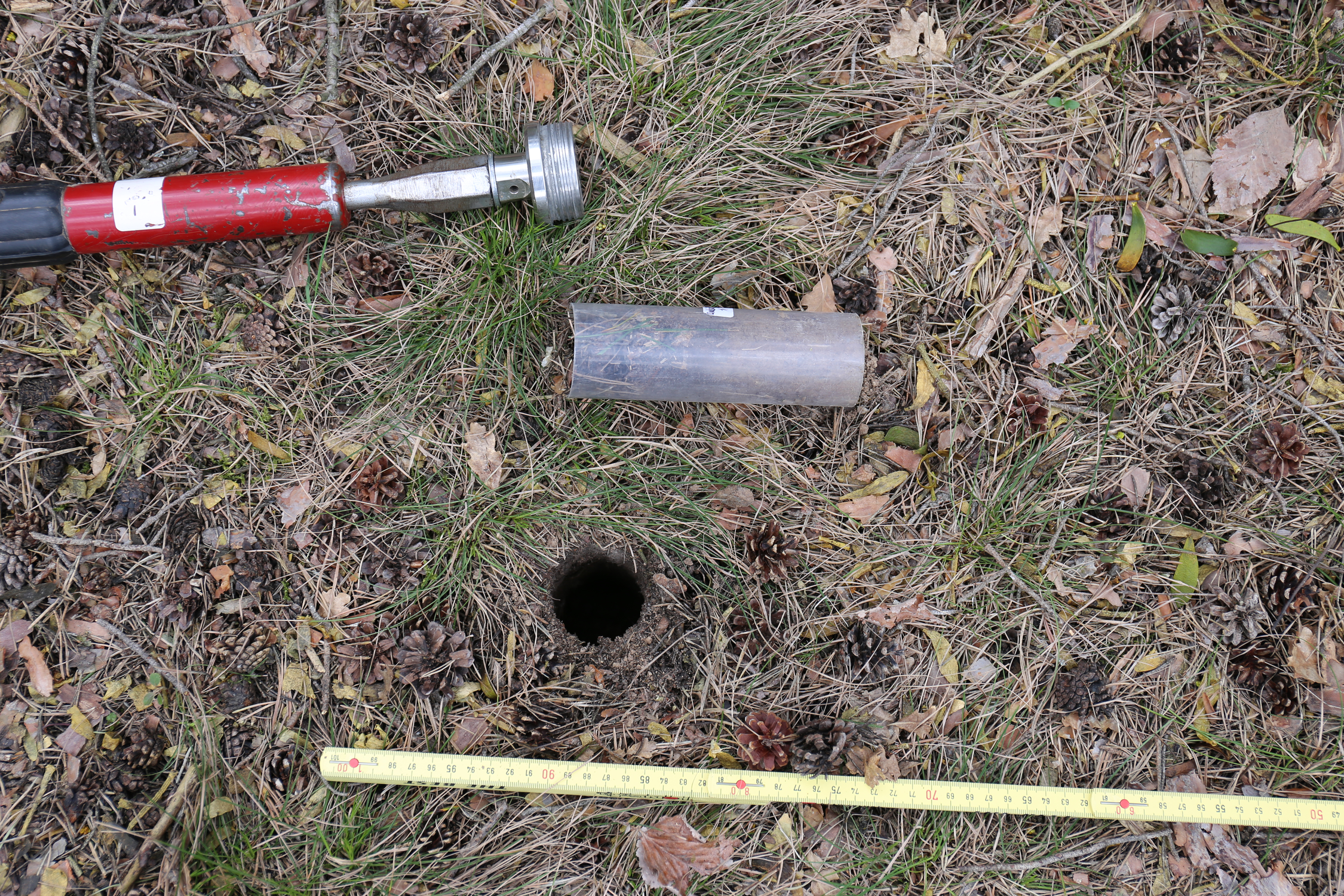

Supplement: S3 Fig — A sample cylinder of 5 cm in diameter and 15 cm length was driven into the soil with a hammer in the vicinity of each acoustic sensor once per season. (TIF) [file pone.0263618.s003.tif]

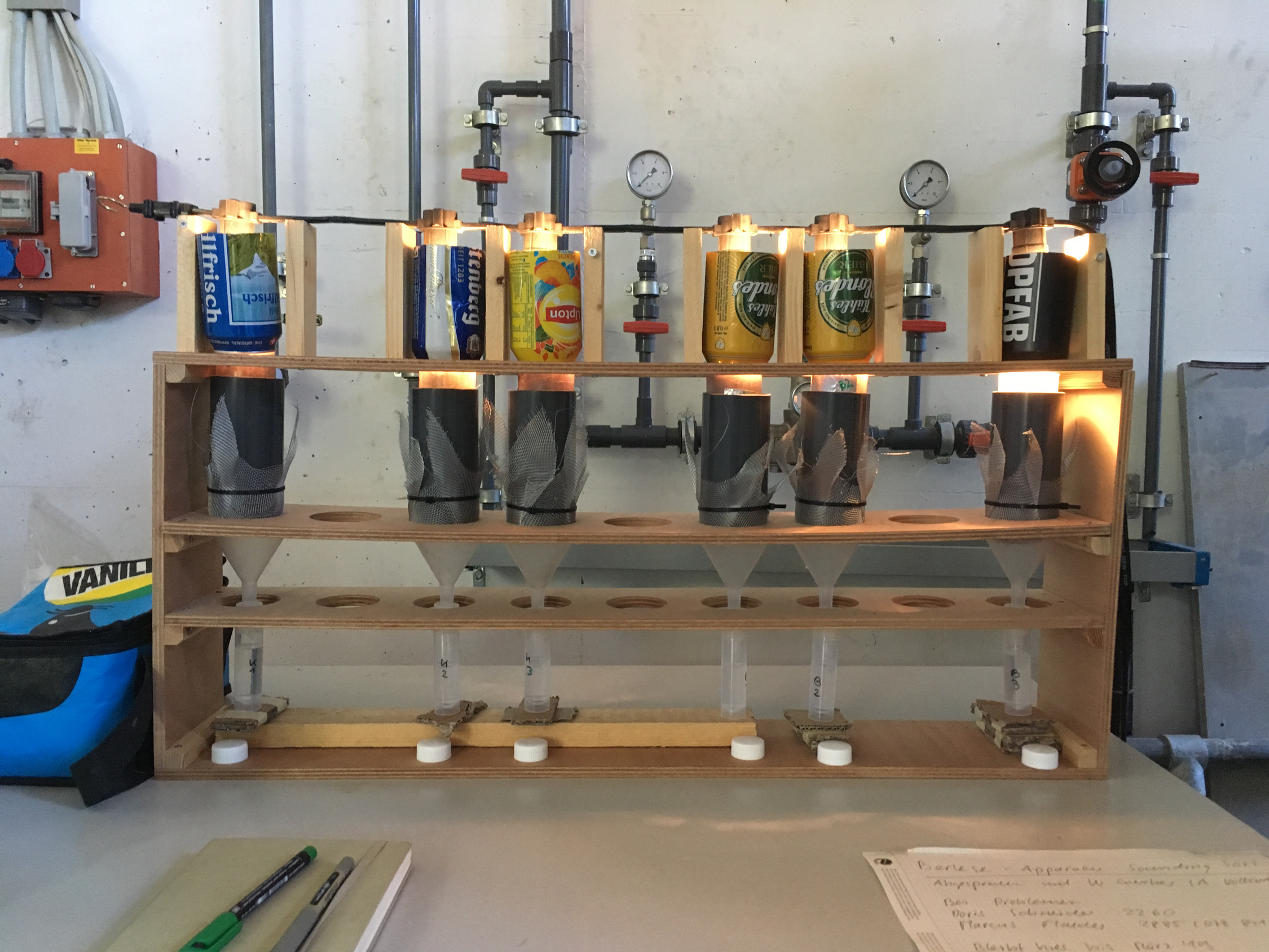

Supplement: S4 Fig — Ordinary light bulbs (40 W) were used and fauna was extracted for 14 days. (TIF) [file pone.0263618.s004.tif]

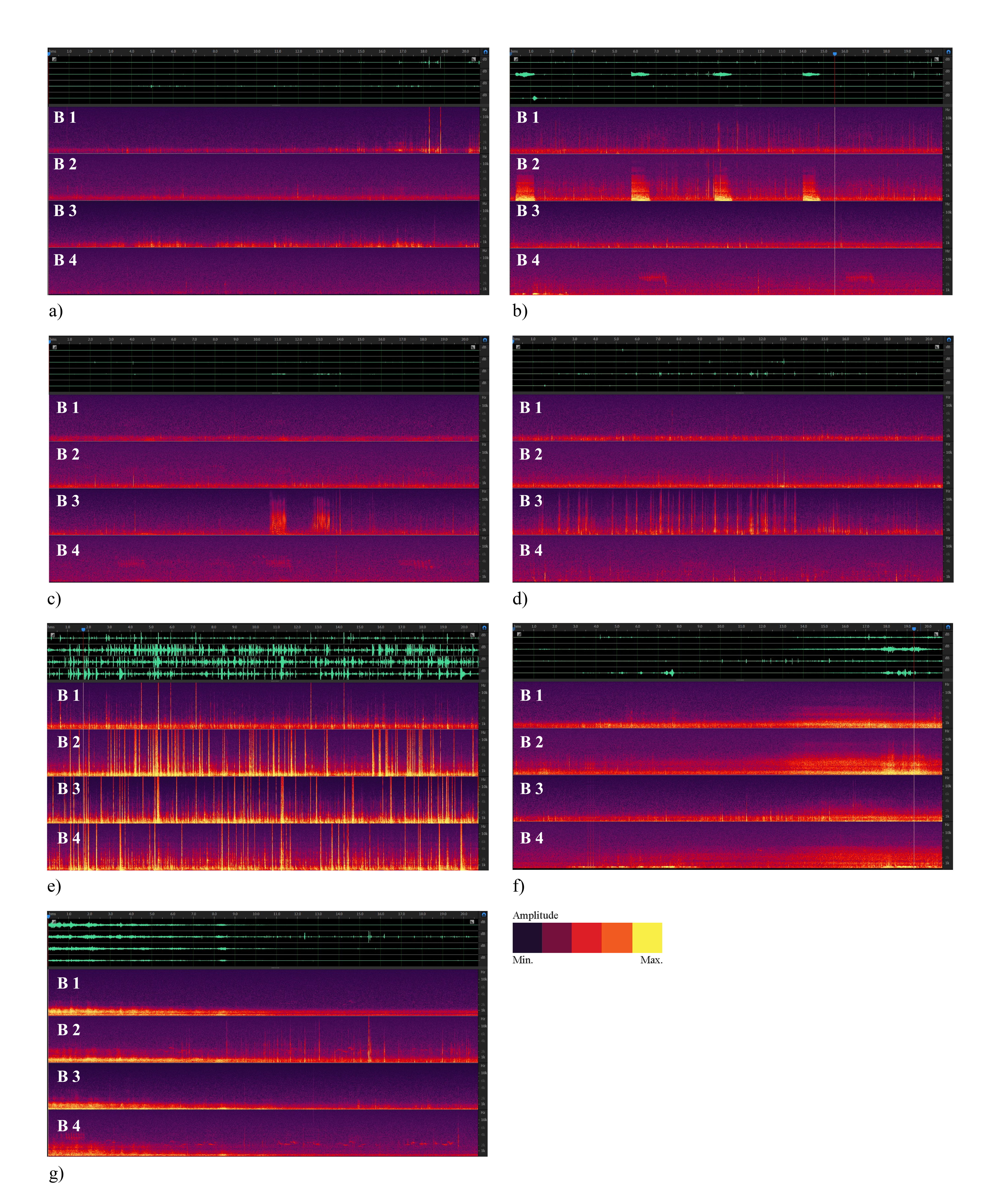

Supplement: S5 Fig — The soil soundscape showed a characteristic band of acoustic emissions between 100 and 1000 Hz. The emissions within this band consisted of a mix of background noises, such as animals moving in greater distance to the sensor (same emissions as close to the sensor, but with lower amplitude), plant root emissions (resulting from a comparison with emission characteristics of plant roots, described by Gagliano et al. [70], or physical sound sources, such as moving pore water and air (resulting from a comparison with signal characteristics of soil structure alterations and waterfront movements, described by Moebius [71] and Flammer et al. [72]. The most significant and loudest sounds seemed to be movement and feeding noises of animals close to the sensor (see S5A and S5D Fig channel 3) in frequency bands between 100 and 10’000 Hz. a) Spectrogram of the acoustic activity in channels B1–4 on 20.06.2019 12:15 (B1–3/CH 1–3, CH 4 is control in the air). The constant emission band on CH 1–3 shows the spectrum of the soil soundscape. On CH 1 and 3, the spectrogram indicates the movement noises of nearby soil animals. b) Sensors B1–4 on 14.06.2019, 15:45. Vibrational calls (CH 2), presumably of a soil insect. Channel 4 in the air shows bird calls. The group of primary decomposers—to a large extent insects and other arthropods that live in and from the litter layer and the uppermost organic soil layers—produce movement and feeding sounds, while the frequencies seem to depend on their body size [73]. Some of these arthropods seem to use the soil matrix as a communication medium. Thus, they produce vibratory sounds with their body or their stridulation apparatus, which propagate over short distances and presumably serve as near-field communication. In the following, a few characteristic examples are highlighted. c) Sensors B1–4 on 18.06.2019, 07:05. Stridulation calls (CH 3), presumably by Myrmica rubra or Myrmica ruginodis (observed at the sensor location). (TIF) [file pone.0263618.s005.tif]

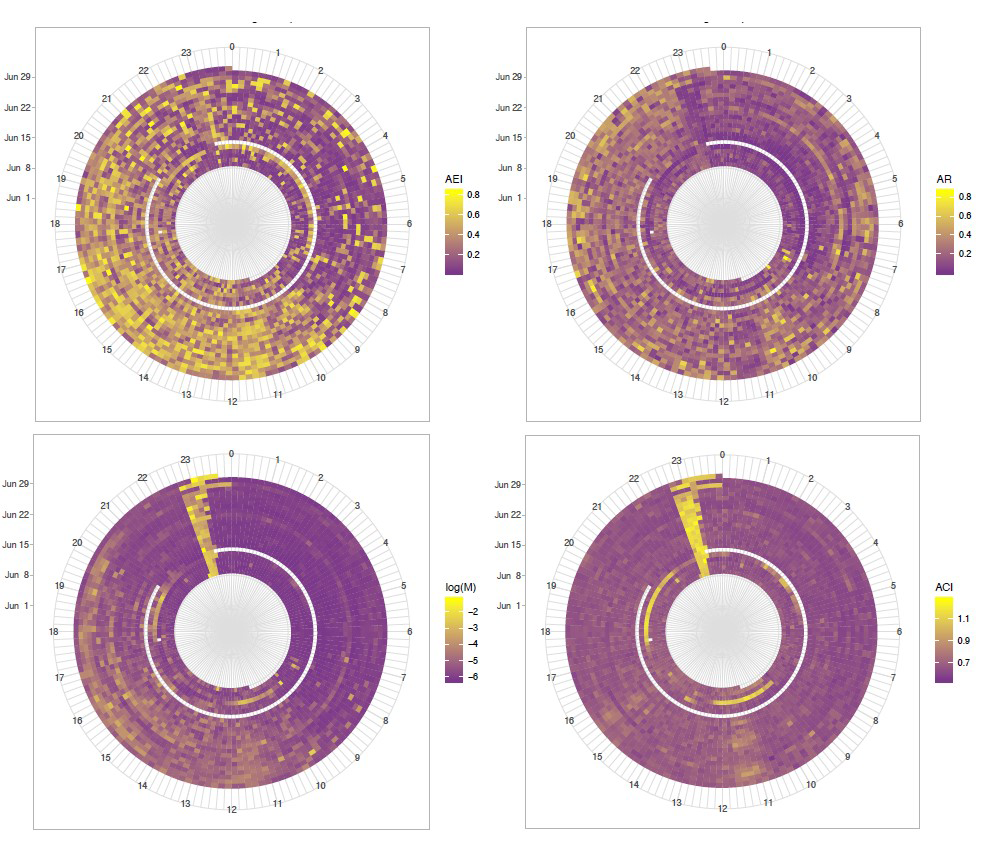

Supplement: S6 Fig — The results of our tests show that no index other than ACI varied over time, and it was even hard to distinguish the daily irrigation periods in the graphs of AEI and AR. Moreover, ACI performed best in resolving daily and seasonal patterns best. To illustrate the temporal dynamics of acoustic complexity at the single measurement spots, heatmaps in a spiral shape were produced. This also allowed detection of specific outstanding events, such as rain or irrigation, in the treatment plot (see S2C Fig). ACI values were mapped with a color scale from dark magenta to light yellow on the spiral graphs below. The higher the ACI, the brighter the color on the spiral graph is represented. Rain and irrigation cause frequent clippings when they hit the acoustic sensors. ACI during rain periods was therefore represented by the brightest colors on the graphs. Similarly, the daily irrigation at the irrigation plot around 23:00 is clearly rendered in yellow. Acoustic diversity in June was recorded by Sensor B1 in the irrigation plot, represented through four acoustic indices: the acoustic evenness index (top left), the acoustic richness (top right), the median of amplitude envelope (bottom left), and the acoustic complexity index (bottom right). Spiral heatmaps of the period 1–29 June 2018; one rotation in the circle represents 24 h. The bright yellow spike shows the irrigation around 23 h. (TIF) [file pone.0263618.s006.tif]

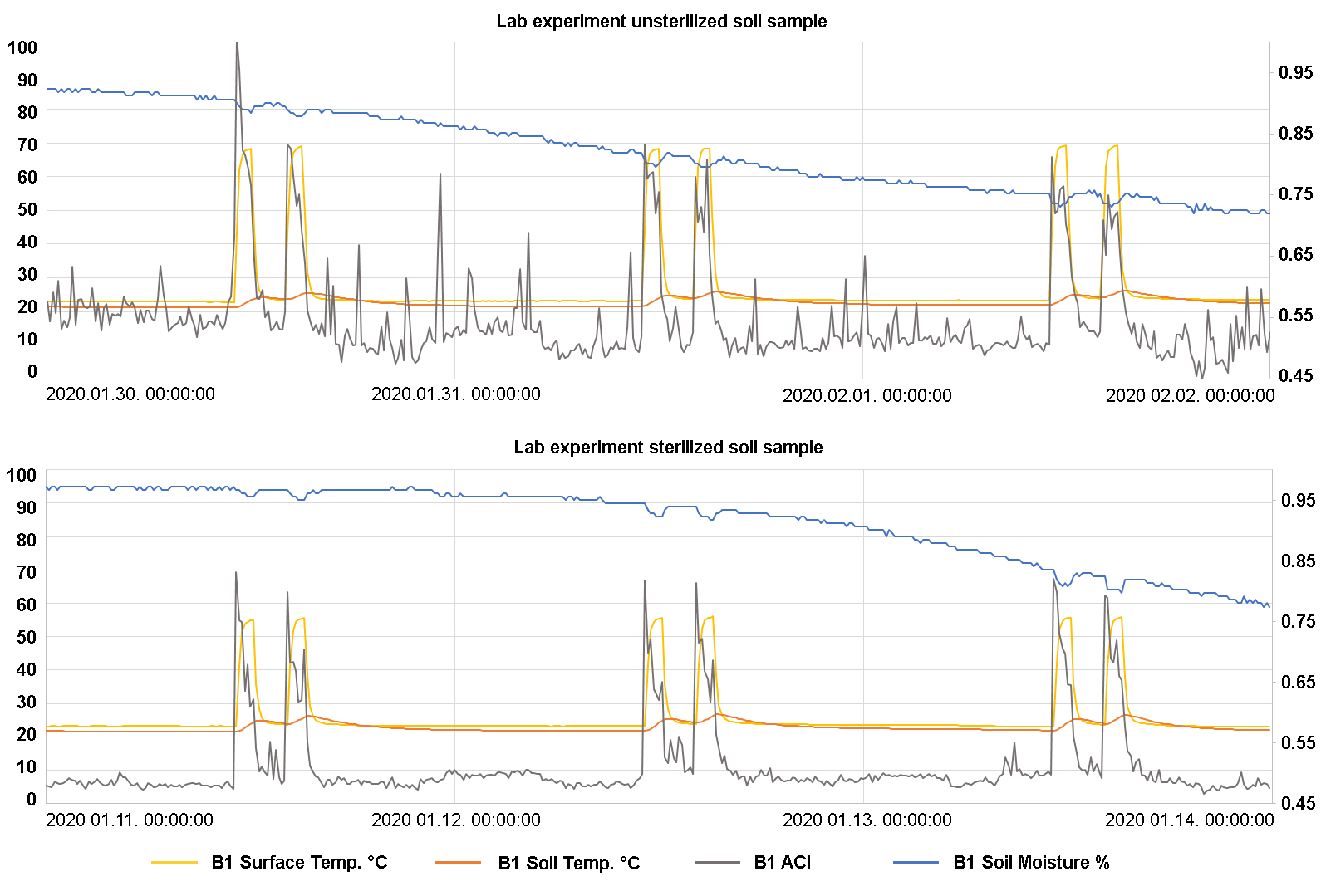

Supplement: S7 Fig — Upper diagram: Untreated sample. Lower diagram: Sterilized sample (frozen at -16° C). Shown here is only one of three samples for each treatment because all samples showed similar patterns. To evaluate whether temperature changes in the soil and on its surface generate acoustic emissions of a biotic or abiotic nature, a laboratory experiment was set up. Three soil samples (puncture samples with a diameter and depth of 10 cm) were collected from the control area in the forest and brought cooled to the laboratory. They were subjected to two 1-h heating cycles under infrared heat lamps in a Faraday cage (see S9 Fig). This was done to imitate the falling of sunlight on the forest floor. The three samples were equipped with the same sensors as in the forest, and three days in a row were recorded and measured (see S7 Fig). The three samples were first exposed to heat untreated directly from the field (see S7 Fig, upper diagram). Then, they were sterilized in the freezer at -16° C. The sterilization process was probably not complete, but we did not want to destroy the sample’s matrix texture by completely freezing it. Individual soil organisms may have survived the freezing process (as the weaker but not completely low ACI curves show in the lower diagram in S7 Fig). The samples were then left to defrost for 48 h and exposed again to the infrared lamps. (TIF) [file pone.0263618.s007.tif]

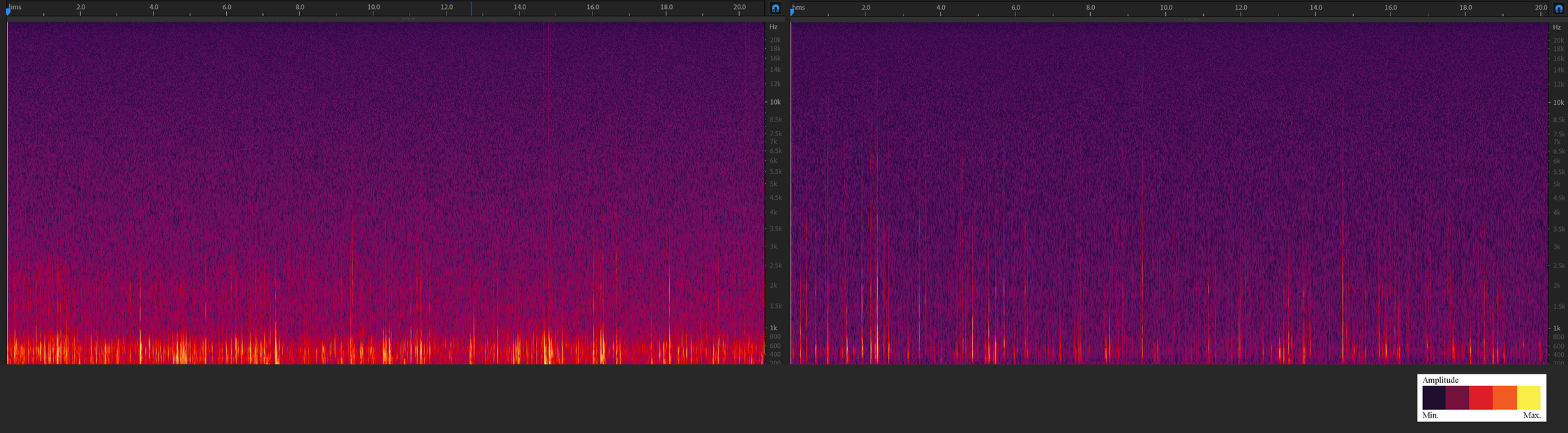

Supplement: S8 Fig — Left: untreated soil sample; right: sterilized soil sample. Short high frequent spikes seem to stem from structural changes or evaporating pore water, while broadband signals between 100–1000 Hz seem to originate from soil life. (TIF) [file pone.0263618.s008.tif]

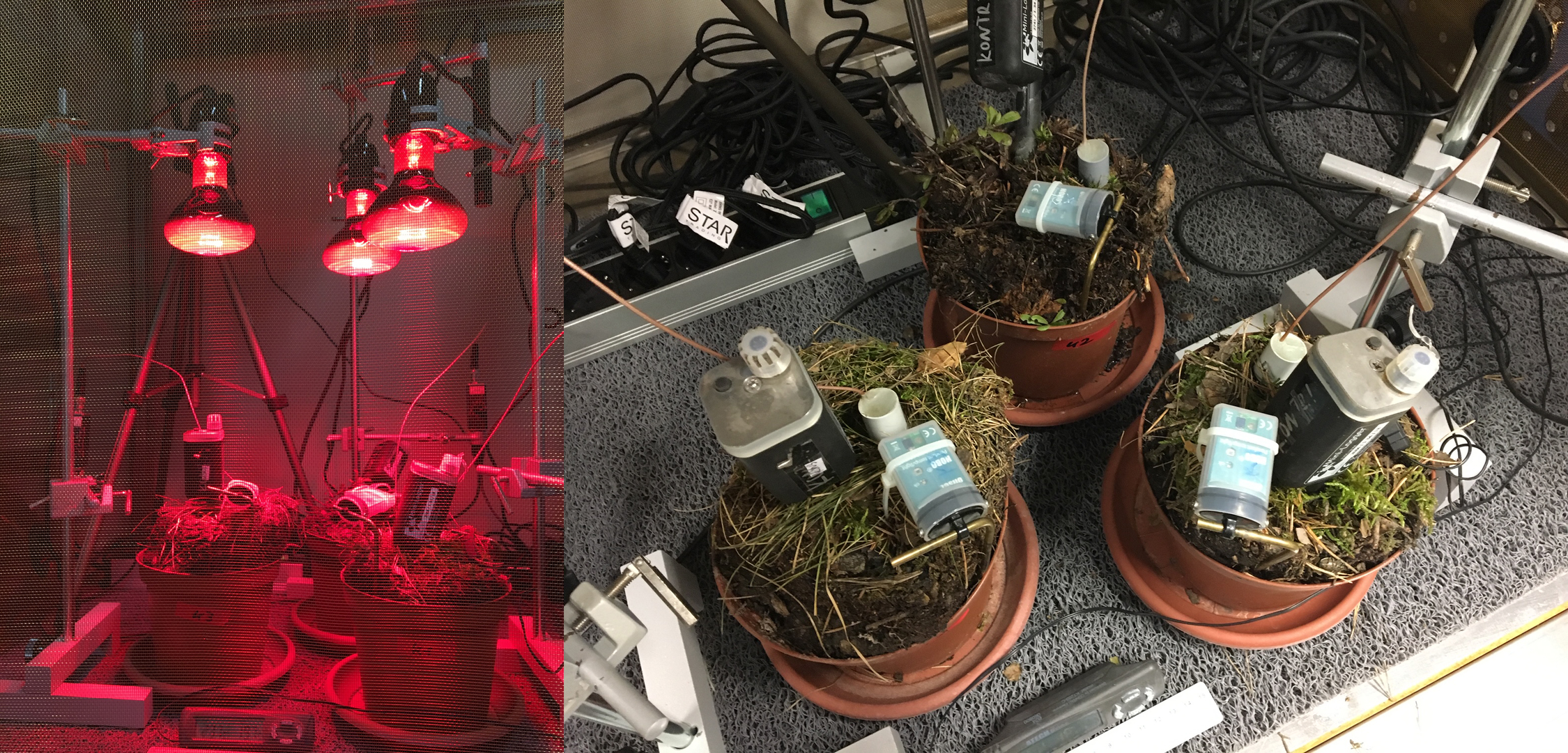

Supplement: S9 Fig — (TIF) [file pone.0263618.s009.tif]

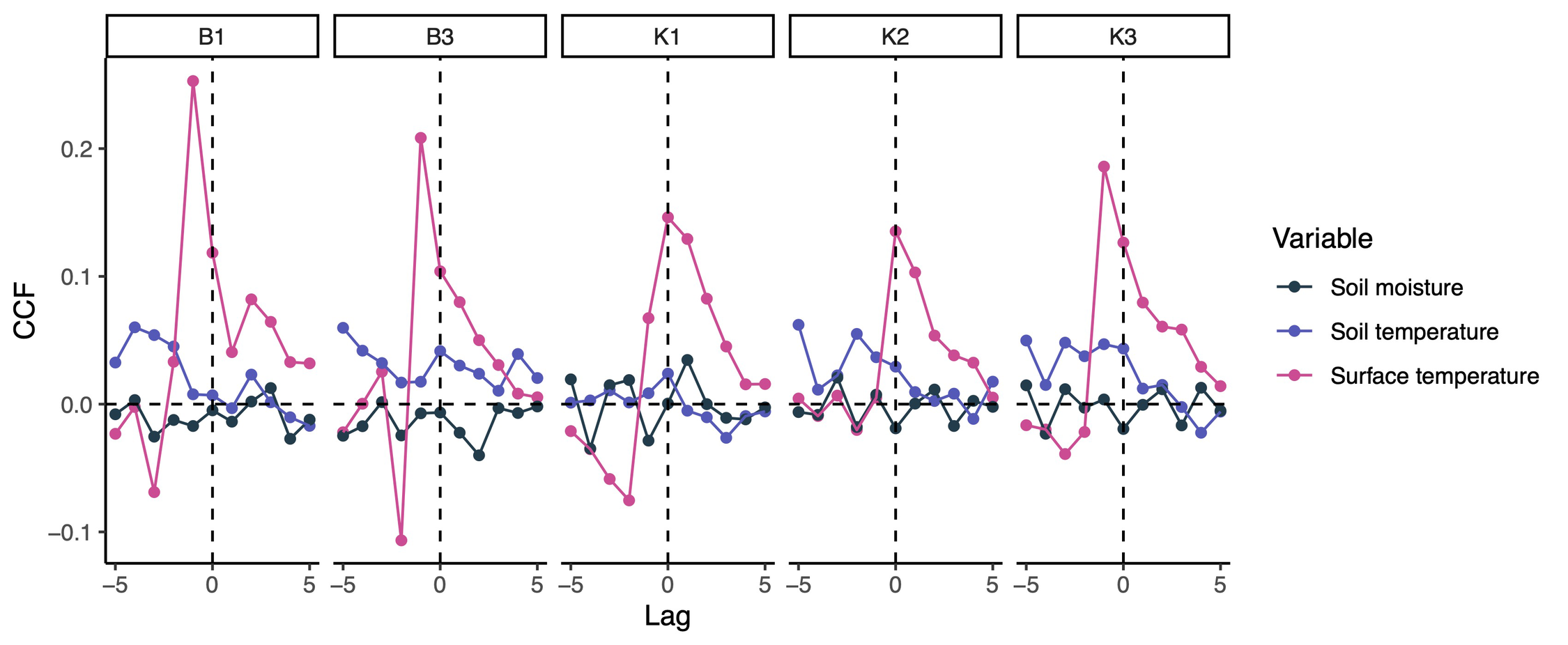

Supplement: S10 Fig — ACI and microclimate variables were differentiated and pre-whitened previously. High correlation at negative lags indicates a strong correlation between ACI and future microclimate, whereas high correlation at positive lags indicates a strong correlation between ACI and past microclimate. (TIF) [file pone.0263618.s010.tif]

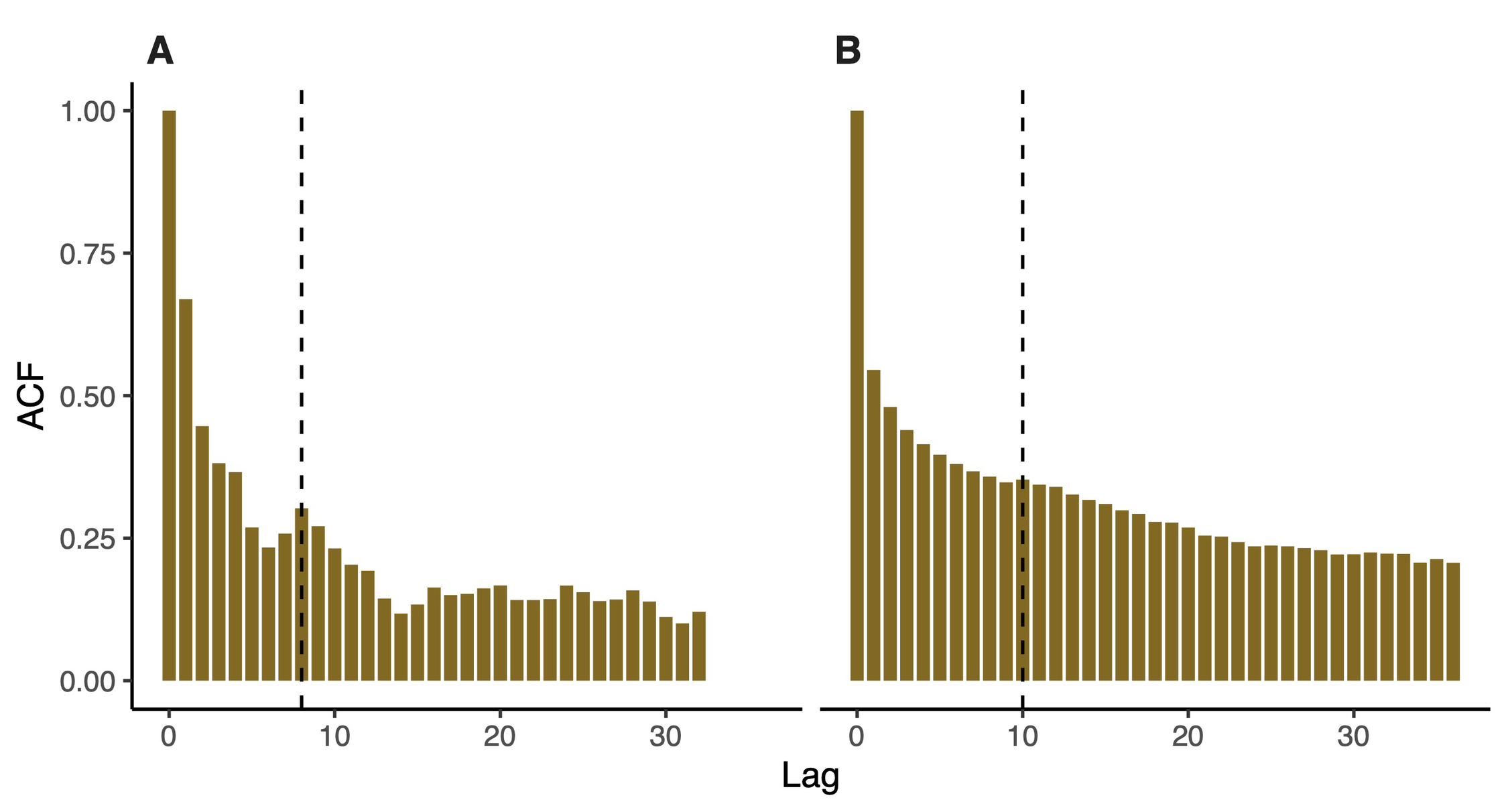

Supplement: S11 Fig — Autocorrelation function (ACF) from residuals of linear mixed-effect models analyzing the effect of (A) daytime and season and (B) microclimate on ACI. Based on these ACFs, the maximum lag was chosen for the autoregressive model in the hierarchical models. Chosen values are indicated by the dashed lines. Note that the time steps of the two models are different (6 h in model A, 10 min in model B). (TIF) [file pone.0263618.s011.tif]

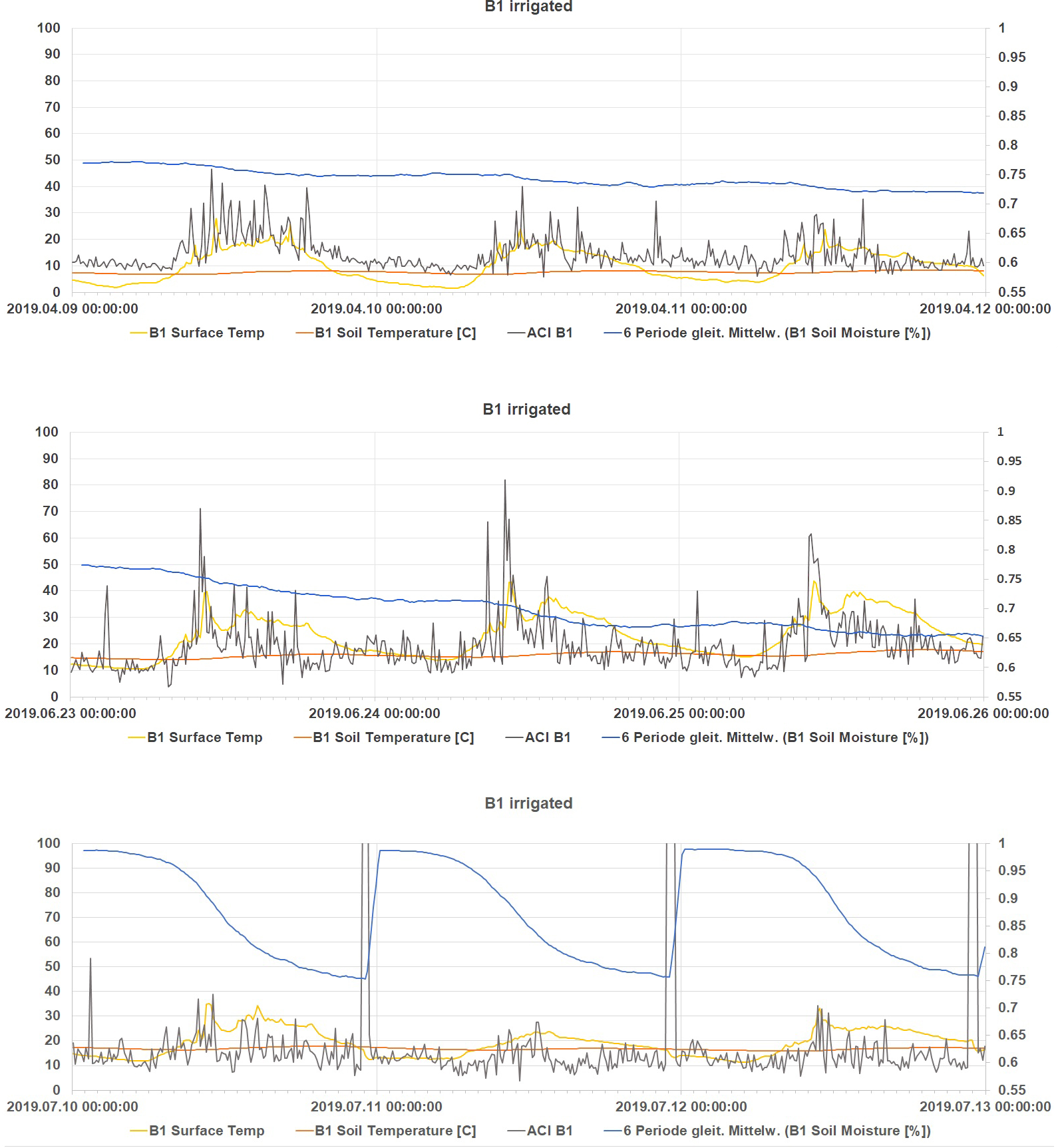

Supplement: S12 Fig — a) Period in spring, 9–12 April 2019; b) period in early summer, 23–26 June 2019; c) period in mid-summer with active irrigation, 10–13 July 2019. (TIF) [file pone.0263618.s012.tif]

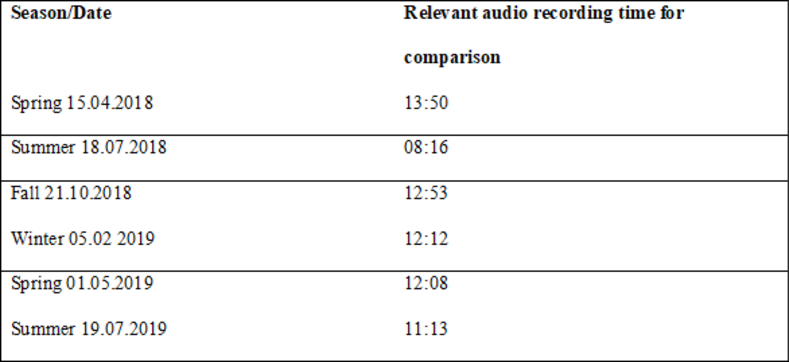

Supplement: S1 Table — Comparison tests showed that it is crucial to select recordings made directly before the soil sampling. This is due to the high dynamics in the activity and composition of local soil fauna. (TIF) [file pone.0263618.s013.tif]

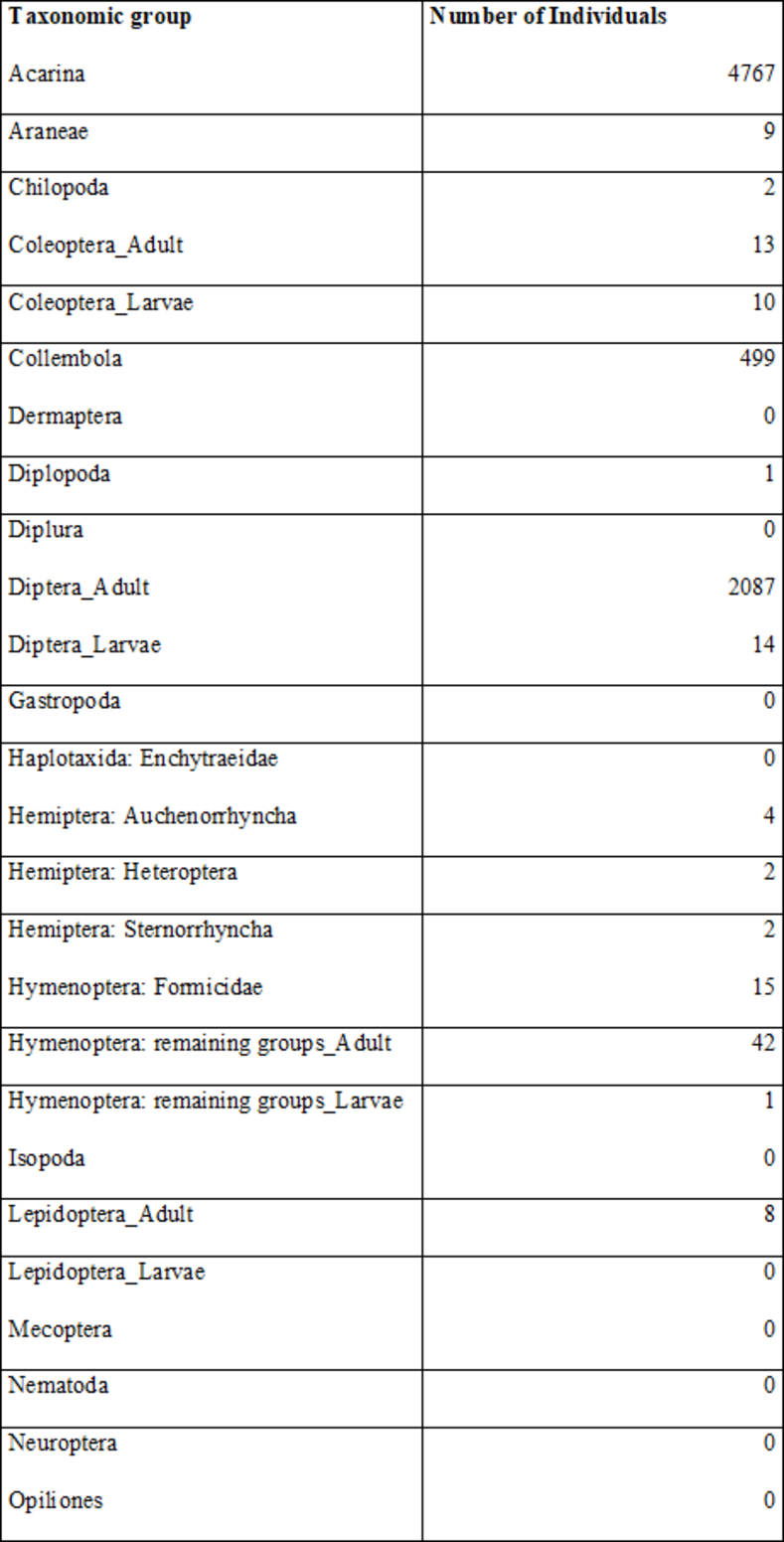

Supplement: S2 Table — Taxa were identified using [67–69]. (TIF) [file pone.0263618.s014.tif]

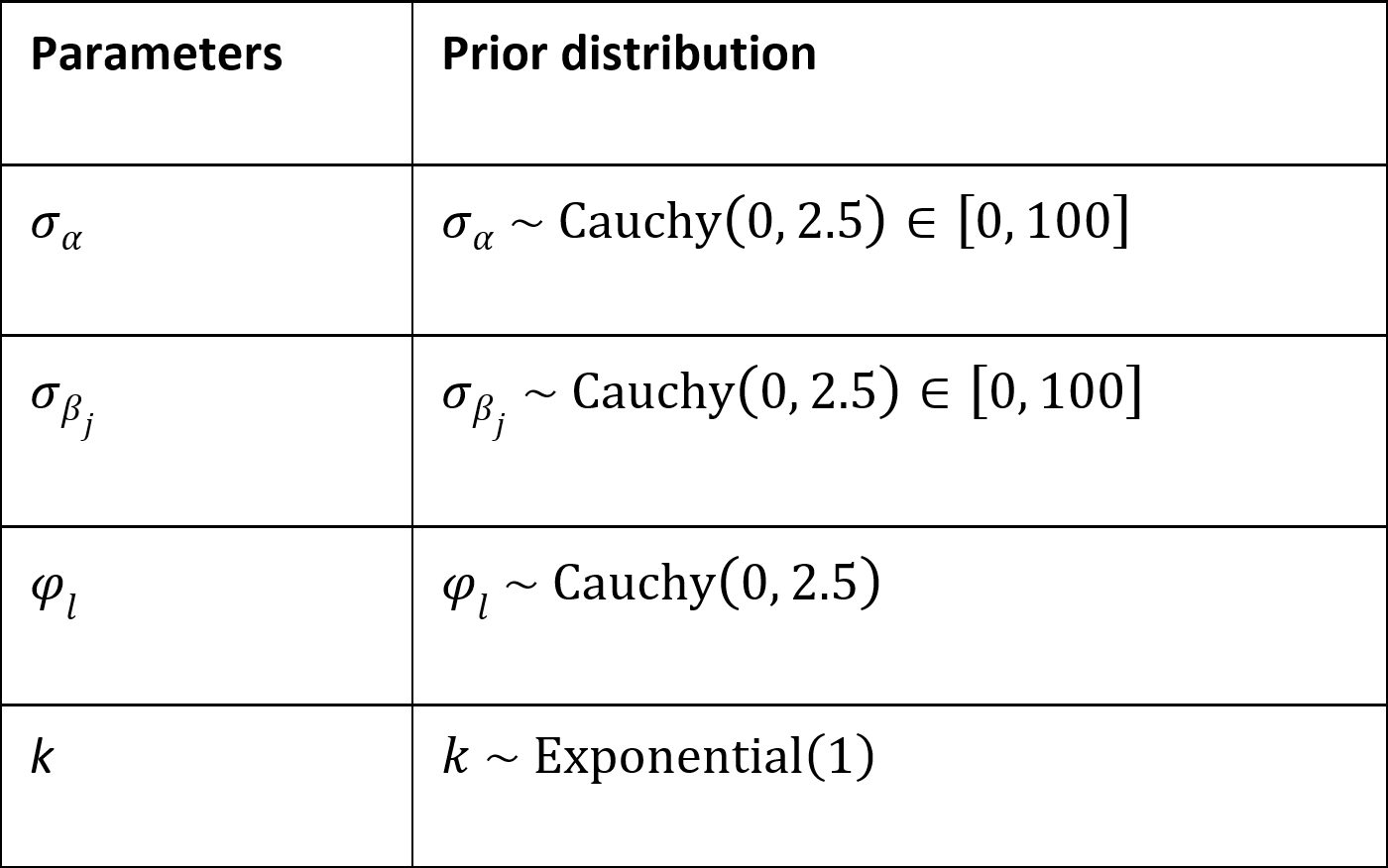

Supplement: S3 Table — (TIF) [file pone.0263618.s015.tif]
